# Factors associated with death coping ability among geriatric nurses and development of culturally sensitive death education interventions

**DOI:** 10.1371/journal.pone.0356462

**Published:** 2026-08-25

**Authors:** Shixiu Liu, Zhijing Li, Yujun Wang, Wenjing Xie

**Affiliations:** 1 Department of Geriatric Respiratory Medicine, The First Affiliated Hospital of Kunming Medical University, Kunming, Yunnan, China; 2 School of Nursing, Kunming Medical University, Kunming, Yunnan, China; Stamford Health System: Stamford Hospital, UNITED STATES OF AMERICA

## Abstract

**Background:**

As population aging accelerates, the demand for high-quality end-of-life care for older adults continues to increase. Nurses’ death coping ability is essential to the delivery of compassionate end-of-life care and to the maintenance of nurses’ psychological well-being. However, in cultural contexts where death is often considered a taboo topic, limited evidence is available regarding geriatric nurses’ death coping ability, its associated factors, and appropriate strategies for death education. This study aimed to assess the level of death coping ability among geriatric nurses, identify associated factors, and develop culturally sensitive strategies for death education.

**Methods:**

A cross-sectional mixed-methods study was conducted in a tertiary hospital in Kunming, China. Using convenience sampling, 203 nurses were recruited from nine geriatric medical wards, three geriatric surgical wards, and a geriatric intensive care unit. Quantitative data were collected using a demographic information questionnaire and the Chinese version of the Coping with Death Scale. Semi-structured interviews were also conducted to explore nurses’ perceptions of death, end-of-life care, and death education. Quantitative data were analyzed descriptively, and qualitative data were examined using thematic analysis.

**Results:**

The mean Coping with Death Scale score was 110.12 ± 24.35, with a standardized score rate of 54.19%, indicating a moderate level of death coping ability. Among the six dimensions, life review ability had the highest score, followed by end-of-life coping ability, death acceptance ability, ability to reflect on and express views about death, ability to discuss one’s own death, and ability to discuss others’ death. The corresponding scores were 33.22 ± 8.765, 18.82 ± 6.698, 18.62 ± 4.203, 15.66 ± 5.057, 14.22 ± 5.735, and 9.58 ± 3.071, respectively. Interview findings showed that traditional cultural beliefs contributed to nurses’ avoidance of death-related conversations. Nurses’ attitudes toward death were also closely related to how they communicated with and cared for terminally ill patients and their families.

**Conclusion:**

Geriatric nurses in this hospital demonstrated a moderate level of death coping ability, which appeared to be closely associated with traditional cultural taboos surrounding death. Death education programs for geriatric nurses should therefore be culturally embedded and systematically designed. Recommended strategies include establishing a dynamic assessment system and developing progressive training modules that address self-acceptance of death, communication skills in end-of-life care, and grief support for family members. Nurses’ years of professional experience, previous exposure to end-of-life care, cultural background, and religious beliefs should be considered when tailoring these interventions. This study provides an empirical basis for developing culturally sensitive death education in the Chinese nursing context. Future randomized controlled trials are needed to evaluate the long-term effectiveness of these strategies.

## Introduction

With the rapid aging of China’s population, the demand for geriatric nursing services is facing unprecedented challenges. As frontline providers of end-of-life care, geriatric nurses are expected not only to alleviate patients’ physical suffering but also to implement comprehensive strategies that help patients adapt to the physical and psychological challenges associated with the dying process [1–7]. In addition to professional nursing knowledge and geriatric care skills, they are responsible for communicating with patients and families about sensitive end-of-life issues, providing dignified care, and collaborating closely with multidisciplinary teams. These responsibilities require competencies in education, communication, and evidence-based practice [8–12]. Geriatric nurses’ death coping ability and attitudes toward death significantly influence their end-of-life care attitudes, care behaviors, and ultimately, patients’ quality of life. These factors are equally critical to nurses’ own physical and psychological well-being and overall quality of life [13]. Therefore, systematically assessing geriatric nurses’ palliative care competence and identifying associated factors can provide an empirical basis for developing targeted training programs and death education strategies, thereby contributing to the improvement of geriatric nursing services.

Previous studies have shown that geriatric nurses who lack adequate competence in coping with and accepting death are more prone to negative emotions and death-avoidant attitudes, potentially perpetuating a maladaptive cycle of negative attitudes toward death [14]. Therefore, it is essential for nurses to deepen their understanding of death as an inevitable process, enhance their death-coping competence, and develop more positive attitudes toward death. These capacities are critical not only for effective emotional regulation and the maintenance of nurses’ psychological well-being, but also for fulfilling their professional responsibilities throughout end-of-life care and the dying process. Further evidence indicates that nurses who have an objective understanding of the nature of death and the value of life are better equipped to address the care needs of terminally ill patients and their families [15,16]. In contrast, nurses with negative attitudes toward death may be more vulnerable to occupational burnout, death anxiety, and avoidance behaviors, and may experience conflicts between their personal and professional roles [17–20]. These difficulties can adversely affect care quality, personal well-being, and physical and psychological health. Collectively, these findings are consistent with clinical observations and provide an empirical rationale for interventions aimed at enhancing death-coping competence among geriatric nurses.

To improve the quality of geriatric nursing services and safeguard nurses’ occupational psychological well-being, numerous studies have examined factors associated with nurses’ death-coping competence. A comprehensive analysis by Zheng et al. identified age, religious belief, work experience, work type, and previous death experiences as key variables associated with this competence [21]. Reference [22] further suggested that higher educational attainment, greater work experience, and stronger professional competence may help buffer stress when nurses are confronted with patient death. Lin et al. reported that among nurses, death-coping self-efficacy was significantly associated with palliative care training, personal bereavement experience, and death attitudes, whereas gender and marital status were not independent predictors in their multivariate model [6]. Confronting patient death often exposes nurses to psychological stress, and the coping strategies they adopt may shape their attitudes toward death and subsequent care behaviors. Prolonged avoidance of death-related distress, persistent work overload, and suppression of emotions may gradually compromise nurses’ physical and psychological health. Over time, these challenges may impair their ability to provide adequate care and emotional support to subsequent patients [13,23]. Notably, support from colleagues and a strong sense of professional identity may reduce nurses’ fear of death, avoidance, and anxiety, thereby contributing to better end-of-life care quality and stronger death-coping competence [24,25].

Nurses’ understanding of end-of-life care, the nature of death, and the value and meaning of life is closely associated with their attitudes toward death. A deeper and more constructive understanding of these concepts may help foster positive attitudes toward death, strengthen death-coping competence, and improve clinical nursing practice. These improvements may, in turn, contribute to higher-quality end-of-life care and support nurses’ physical and psychological well-being. Conversely, nurses who lack an accurate understanding and acceptance of death may experience persistent death-related distress, making clinical practice a continuing source of psychological burden. Accordingly, nurses need not only the professional competencies required to care for terminally ill patients but also a scientifically informed and constructive perspective on life and death. Systematic death education represents an important means of achieving this goal. Previous studies have shown that death education can improve nurses’ attitudes toward death and enhance their death-coping competence [11,13,18,26]. However, in cultural contexts where death is traditionally regarded as a taboo topic, death-related communication remains highly sensitive. In clinical practice, illness concealment by patients or family members [27–29] and emotional distress among patients and families [30–32] may hinder effective nurse–patient communication about end-of-life issues. These barriers may further constrain nurses’ end-of-life care competence, death-related knowledge, and positive attitudes toward death [30–32]. Therefore, culturally appropriate death education strategies are needed to improve the effectiveness of interventions designed to strengthen nurses’ death-coping competence.

This study was conducted within the framework of traditional Chinese culture to empirically assess death-coping competence among geriatric nurses and systematically examine its relationships with death education needs and attitudes toward death. The study also aimed to identify nurses’ specific needs in caring for terminally ill patients and managing grief-related emotions. Considering the attitudes and cultural backgrounds of patients and their families, culturally appropriate death education strategies and targeted training approaches tailored to the regional context were further explored. These strategies may help reduce nurses’ risk of emotional distress, occupational burnout, and other adverse psychological responses, while strengthening their engagement in end-of-life care.

## Materials and methods

### Study design and participants

This cross-sectional study was conducted from May to June 2025 in a tertiary Grade A hospital in Kunming, China. Participants were recruited by convenience sampling from nine geriatric medical wards, three geriatric surgical wards, and one geriatric intensive care unit.

Inclusion criteria: ϕRegistered nurses who had worked in geriatric departments for at least one year; κNurses who had cared for terminally ill patients within the previous six months; λNurses who voluntarily participated in the study and provided written informed consent.

Exclusion criteria: ϕQuestionnaires with more than 20% missing items; κQuestionnaires showing regular or patterned responses; λTrainee nurses undergoing clinical training, and nurses who were absent during the survey period.

The study included 17 variables. According to the sample size estimation method for multivariable analysis [33], at least 10 participants were required per independent variable. Accounting for an expected 10% invalid response rate, the minimum required sample size was 189 participants. Ultimately, 203 valid questionnaires were collected. For the qualitative component, purposive sampling was employed to recruit 11 nurses for semi-structured in-depth interviews, designated as N1–N11. Participants were selected to maximize variation in gender, age, marital status, religious belief, educational level, professional title, and years of geriatric nursing experience. Recruitment continued until data saturation, when no new themes emerged.

### Instruments

**General information questionnaire:** The questionnaire was constructed through literature review and expert consultation, referencing the validated Death Attitude Profile Scale and Death Education Needs Scale, together with a general information form. This study selected 14 sociodemographic variables that may affect the death coping ability of geriatric nurses, including age, gender, educational background, marital status, ethnicity, professional title, and working years, whether they participated in death education and training, and whether they participated in end-of-life management of patients, etc.

**Death-coping self-efficacy scale (DCSS):** The Coping with Death Scale was developed by the American scholar Professor Bugen [34], introduced and translated into Chinese by Chinese scholars Zheng Ruishang and Guo Qiaokhong in 2019 [35]. The scale used in this study comprises the following six dimensions, totaling 28 items: the ability to communicate with others about the end of life or death (8 items), the ability to accept death (5 items), the ability to deal with matters after death (4 items), the ability to cope with death (4 items), the ability to reflect on and express one’s own mortality (5 items), and the ability to live life (2 items). A 7-point Likert scale was used, where 1–7 points correspond to “strongly disagree” (1 point), “neutral” (4 points), to “strongly agree” (7 points). Item 12 is the reverse score; the sum of the scores for each item is the total score, which ranges from 28 to 196. The higher the score, the better the subject’s ability to cope with death. The Cronbach’s alpha coefficient was 0.905, the reliability was 0.973, and the content validity was 0.99.

### Data collection

#### Questionnaire survey.

After providing informed consent, participants received standardized instructions regarding the study purpose and questionnaire completion procedures. Following brief unified training, participants completed the questionnaires anonymously and independently within 8–15 minutes. Completed questionnaires were collected immediately and screened for validity before coding and entry into a computerized database for quantitative analysis. A total of 220 questionnaires were distributed, and 203 valid questionnaires were returned, yielding an effective response rate of 92.27%.

#### Semi-structured interviews.

A descriptive qualitative design was adopted to explore geriatric nurses’ experiences in coping with patient death through face-to-face, semi-structured interviews. The study was reported in accordance with the Consolidated Criteria for Reporting Qualitative Research (COREQ). Interviews were conducted in Mandarin Chinese in quiet private settings, such as ward meeting rooms, and lasted approximately 35–50 minutes. Before each interview, the researcher explained the study’s purpose and obtained informed consent. All interviews were audio-recorded with permission, supplemented by field notes. Probing, clarification, and reflective questioning were used to explore participants’ responses further when necessary.

The interviews focused on five topics: experiences caring for dying patients within the previous six months; the impact of these experiences on daily life; attitudes toward death and end-of-life care; understanding of death and end-of-life care; and challenges and coping strategies related to death care. Example questions included: “Please describe your recent experience caring for a terminally ill patient”; “How has this experience affected your daily life?”; “What challenges do you face in death care, and what strategies may help address them?”; “Do you discuss death or death-care experiences with family or friends?”; “Have you received death education training, and how has it influenced your ability to cope with death?”; and “How do you or your colleagues manage conflicts between end-of-life care responsibilities and personal life?”.

### Ethical considerations

This study was approved by the Ethics Committee of the First Affiliated Hospital of Kunming Medical University (approval no. 2025L no.127) and conducted in accordance with relevant institutional and local ethical requirements. Written informed consent was obtained from all participants prior to enrollment. All participant information was kept strictly confidential and used solely for research purposes. To protect privacy, identifiable information was replaced with coded identifiers in all study records and publications.

### Quality control

All researchers received standardized training before data collection and analysis to ensure consistency in study objectives, questionnaire content, and survey procedures. Standardized instructions were used during questionnaire administration. Completed questionnaires were collected immediately and checked for completeness and accuracy, and participants were asked to correct missing or unclear responses when necessary. During the interviews, leading or suggestive questions were avoided, and unclear statements were clarified promptly. Interview summaries were reviewed with participants for confirmation after each session. Participants’ emotional responses were monitored throughout the interviews. If a participant showed discomfort or reluctance to continue, the interview was terminated immediately, and another participant was recruited.

### Data analysis

Interviews were transcribed verbatim immediately after each session. Content analysis was performed through iterative transcript review, thematic framework development, statement coding, theme refinement, and participant verification of findings. Two researchers independently conducted coding and analysis, and disagreements were resolved through discussion within the research team. Data were organized and exported using Microsoft Excel and analyzed with SPSS version 23.0. Descriptive statistics, including means, standard deviations, frequencies, and percentages, were used to summarize participants’ demographic characteristics and Coping with Death Scale scores. Independent-samples *t* tests, one-way analysis of variance (ANOVA), and post hoc multiple comparisons were performed to examine differences across demographic groups, including sex, age, professional title, and department. Multiple linear regression analysis was conducted to identify factors associated with death-coping competence. All statistical tests were two-sided, and *p* < 0.05 was considered statistically significant.

## Results

### Participant characteristics

A total of 203 geriatric nurses were included, including 186 females (91.63%) and 17 males (8.37%). Among them, 105 (51.72%) were married, 96 (47.29%) were unmarried, and 2 (0.99%) were divorced. Participants ranged in age from 21 to 50 years, with a mean age of 29.82 ± 5.42 years. Work experience ranged from 1 to 32 years, with a mean of 7.33 ± 6.31 years, and 73.1% had less than 10 years of experience. Most participants held a bachelor’s degree (95.07%), while 2.96% held a master’s degree and 1.97% had a secondary vocational nursing qualification. In addition, 93.60% reported no religious affiliation, 76.85% had not received death education or related training, and 77.83% had experience caring for terminally ill patients.

### Current situation of death coping ability of geriatric nursing nurses

The Chinese version of the Coping with Death Scale (DCS) used in this study consisted of 28 items across six dimensions: ability to reflect on and express views about death (8 items), ability to discuss one’s own death (5 items), ability to discuss others’ death (5 items), death acceptance ability (4 items), end-of-life coping ability (4 items), and life review ability (2 items). Detailed scores for each dimension are presented in Table 1, and the five highest- and five lowest-scoring items are shown in Table 2.

**Table 1:** The death coping ability score of geriatric nurses (n = 203).

items	total score (*M*±*SD*)	item average score (*M*±*SD*)
Life review ability	33.22±8.77	4.15±1.10
End-of-life coping ability	18.82±6.70	3.76±1.34
Ability to discuss one’s own death	14.22±5.74	3.56±1.44
Ability to reflect on and express views about death	15.66±5.06	3.92±1.27
Death acceptance ability	18.62±4.20	3.72±0.84
Ability to discuss others’ deaths	9.58±3.07	4.79±1.54
Total score	110.12±24.35	3.93±0.87

**Table 2:** Five highest- and lowest-scoring items on the death coping ability scale (n = 203).

items	item score (*M*±*SD*)	ranking
The five highest scoring items		
The quality of my life is more important than the length of my life	4.86±1.01	1
I’m making the most of my life right now	4.71±1.07	2
Before I or someone else dies, I can tell them how much I love them	4.63±1.03	3
Do I know how to listen to others, including the dying	4.54±0.97	4
If I need to, I will spend time with dying patients	4.39±0.87	5
The five lowest-scoring items		
I am prepared to face death	3.49±1.05	24
I am prepared for end-of-life care	3.29±1.12	25
I am familiar with the various arrangements for a funeral	3.26±1.08	26
I understand the services offered by funeral homes	3.22±1.13	27
I have a clear plan in place for death and the end of life	3.07±0.98	28

The mean total DCS score among the 203 geriatric nurses was 110.12 ± 24.35, corresponding to a standardized score rate of 54.18%, indicating a moderate level of death-coping competence. Among the six dimensions, life review ability had the highest score (33.22 ± 8.765), followed by end-of-life coping ability (18.82 ± 6.698), death acceptance ability (18.62 ± 4.203), ability to reflect on and express views about death (15.66 ± 5.057), ability to discuss one’s own death (14.22 ± 5.735), and ability to discuss others’ death (9.58 ± 3.071). The five items with the lowest scores were “*I am prepared to face death*,” “*I am prepared for end-of-life care*,” “*I am familiar with the various arrangements for a funeral*,” “*I understand the services offered by funeral homes*,” and “*I have a clear plan in place for death and the end of life*.” The results of this analysis indicate that the geriatric nurses at the facility lacked a clear understanding of the management of patient deaths and that they were unfamiliar with funeral ceremonies and the range of services offered by the funeral home, which may have affected the quality of their care and nursing behaviors.

### Multiple linear regression analysis of death-coping competence among geriatric nurses

#### Univariate analysis.

Independent-samples t tests and one-way analysis of variance (ANOVA) were used to compare total death-coping competence scores across demographic and occupational characteristics. As shown in Table 3, significant differences were found according to age (*F* = 2.704, *p* = 0.047), educational level (*F* = 2.612, *p* = 0.031), years of geriatric nursing experience (*F* = 4.774, *p* = 0.003), department (*F* = 5.686, *p* = 0.004), professional title (F = 7.393, *p* = 0.001), and previous participation in death education or related training (*t* = 4.533, *p* = 0.034) (all *p* < 0.05). No significant differences were observed according to sex, marital status, religious affiliation, or previous involvement in end-of-life care for relatives or friends (all *p* > 0.05).

**Table 3:** Univariate analysis of geriatric nurses by demographic characteristics (n = 203).

items	N(%)	Score for coping with death	statistics	P
Sex			0.853	0.395
Male	17(8.37)	114.94±22.17		
Female	186(91.63)	109.68±24.55		
age			2.704	0.047
≤30	122(60.10)	105.50±23.50		
31 ~ 40	52(25.61)	112.30±24.10		
≥41	29(14.29)	118.60±25.20		
Marital status			0.291	0.748
Single	96(47.29)	109.52±19.74		
Married	105(51.72)	111.23±27.71		
Divorced	2(0.99)	103.50±14.85		
Religious belief			0.206	0.837
Yes	13(6.4)	111.38±23.19		
No	190(93.60)	109.95±24.42		
Education level			2.612	0.031
College	4(1.97)	102.50±20.50		
Bachelor’s degree	193(95.07)	110.00±24.10		
Master’s degree	6(2.96)	122.50±25.80		
Nursing age			4.774	0.003
1 ~ 5	105(51.70)	109.02±18.33		
6 ~ 10	53(26.10)	107.36±19.38		
11 ~ 15	15(7.40)	121.40±15.36		
≥16	30(14.80)	120.13±22.73		
Professional title			7.393	0.001
The nurse	33(16.30)	108.12±17.44		
Nurse practitioner	123(60.60)	108.37±19.25		
Nurse in charge or above	47(23.20)	120.51±19.62		
Work department			5.686	0.004
Internal medicine	135(66.50)	107.45±23.68		
surgical	42(20.70)	109.81±21.49		
ICU	26(12.80)	124.62±27.58		
Have you received death education or training?			4.533	0.034
Yes	47(23.20)	116.70±23.50		
No	156(76.80)	108.16±24.29		
Have you ever participated in end-of-life care?			1.872	0.051
Yes	105(51.72)	113.19 ± 24.24		
No	98(48.28)	106.83 ± 24.16		

#### Multiple linear regression analysis.

Multiple linear regression was used to identify factors independently associated with death-coping competence among geriatric nurses, with the total death-coping competence score entered as the dependent variable. Variables significant in the univariate analysis—age, educational level, years of geriatric nursing experience, department, professional title, and previous participation in death education or related training—were entered into the model (*α*_In_ = 0.05, *α*_out_ = 0.10). Age, educational level, department, professional title, and years of geriatric nursing experience were treated as continuous variables; participation in death education or training was treated as a binary variable (0 = no, 1 = yes).

As shown in Table 4, the model was statistically significant (*F* = 8.94, *p* < 0.001), with an adjusted *R*^2^ of 0.202, indicating that the included variables explained 20.2% of the variance in death-coping competence. All variance inflation factors were less than 2, indicating that there was no significant multicollinearity.

**Table 4:** Multiple linear regression analysis of geriatric nurses’ death coping ability (n = 203).

The independent variables	B	SE	β	t	P
Constant	68.23	7.89	--	8.65	<0.001
Age	0.48	0.21	0.131	2.29	0.023
Education level	7.89	3.87	0.140	2.04	0.043
Work department	4.23	1.56	0.178	2.71	0.007
Professional title	6.78	1.89	0.241	3.59	<0.001
Nursing age	−0.45	0.21	−0.143	−2.14	0.033
Received any education or training	7.23	3.34	0.136	2.16	0.032

Note: B, unstandardized regression coefficient; SE, standard error; β, standardized coefficient; t, t-value. VIF < 5 for all predictors. R^2^ = 0.227, After the adjustment R^2^ = 0.202, F = 8.94, P < 0.001.

Consistent with the Pearson correlation analysis, age (*β* = 0.131, *p* = 0.023), educational level (*β* = 0.140, *p* = 0.043), department (*β* = 0.178, *p* = 0.007), professional title (*β* = 0.241, *p* < 0.001), years of geriatric nursing experience (*β* = −0.143, *p* = 0.033), and previous participation in death education or training (*β* = 0.136, *p* = 0.032) were independently associated with death-coping competence. Age, educational level, department, professional title, and death education experience were positively associated with death-coping competence, whereas years of geriatric nursing experience showed a negative association. This negative association may reflect emotional exhaustion and occupational burnout related to prolonged exposure to end-of-life care. These findings suggest that interventions for junior nurses should focus on knowledge, skills training, and psychological resilience, whereas those for senior nurses should emphasize emotional support and burnout prevention.

#### Semi-structured interview findings.

To further explore geriatric nurses’ lived experiences and perspectives on coping with patient death, 11 nurses who had participated in the questionnaire survey were purposively selected for face-to-face, semi-structured in-depth interviews. Participants were coded as N1-N11. Their demographic characteristics are presented in Table 5.

**Table 5:** Characteristics of participants.

Participant	Age (Years)	Gender	Education Level	Years of Geriatric Nursing Experience (Years)
N1	48	Female	College	16
N2	42	Female	College	11
N3	29	Female	Bachelor’s degree	5
N4	26	Female	Bachelor’s degree	2
N5	27	Female	Bachelor’s degree	3
N6	31	Female	Master’s degree	4
N7	35	Female	Bachelor’s degree	8
N8	26	Female	Bachelor’s degree	3
N9	34	Male	Master’s degree	5
N10	25	Female	Bachelor’s degree	2
N11	32	Male	Bachelor’s degree	7

The interview data were analyzed using thematic analysis, and several key themes were identified, as described in the following.

Social, religious, cultural, and economic factors jointly shape individuals’ attitudes toward death [36]. In this study, nurses’ understanding of death was closely associated with their death-coping competence and was strongly influenced by traditional cultural beliefs. Many nurses, particularly early in their careers, reported avoidance of death-related topics because death was often perceived as inauspicious or emotionally distressing.

*N5: “I think death is frightening. It means bad luck and misfortune…”*

*N8: “I was afraid to talk about death-related issues. During my internship, I tried to avoid end-of-life care whenever possible…”*

*N9: “I do not think death is mysterious, but I see it as a sad topic. Talking about it makes me feel upset.”*

Although sociocultural beliefs shaped nurses’ initial perceptions, long-term clinical practice appeared to gradually reshape their attitudes toward death. Repeated exposure to patient death required nurses to develop end-of-life care skills and emergency response abilities, which helped some nurses move from avoidance toward acceptance.

*N1: “I have worked in nursing for many years and have been involved in many end-of-life care situations. I am no longer afraid… I think death is very normal.”*

*N2: “We gradually accept that death is an inevitable part of our work. I review the care process repeatedly so that I can remain calmer when providing death-related care.”*

These findings suggest that clinical practice may play an important role in helping nurses reconstruct their attitudes toward death.

End-of-life care and patient death often cause negative emotions, including anxiety, fear, sadness, guilt, and frustration, which could affect nurses’ daily lives and psychological well-being.

*N4: “When a patient dies during my shift, I feel lost and unsuccessful. This feeling can last for a long time, affect my daily life, and even influence how I interact with my family…”*

*N6: “Experiences with death care made me feel depressed, and I often had nightmares…”*

*N7: “When I had just graduated, the sadness and loss caused by death care significantly affected my daily life. After experiencing more cases, I learned to separate my personal life from work and tried not to think about it after going home.”*

To cope with this emotional burden, nurses gradually developed boundary-setting strategies. Some limited death-related experiences in the workplace through symbolic practices, such as changing uniforms after work or avoiding work-related discussions at home, to maintain a psychological boundary between professional and personal life.

Effective communication is essential for maintaining trust between nurses, patients, and families [37]. However, the sensitivity of death-related topics created substantial challenges in end-of-life communication.

*N3: “It is difficult to deliver bad news gently. Families often do not want to accept it, and seeing their pain is distressing…”*

*N6: “The way you say it is very important… You have to find the right words…”*

*N10: “I often have difficulty communicating with dying patients. I do not know how to talk with patients or their families…”*

Geriatric end-of-life care also requires multidisciplinary collaboration. Effective teamwork can support continuity and comprehensiveness of care.

*N11: “We are a team, and teamwork ensures that patients are protected in all aspects. The key is to make sure patients receive the best possible care from every perspective.”*

These findings highlight the complexity of communication in palliative and end-of-life care and indicate that nurses need both empathic communication skills and professional competence.

Frequent exposure to death and the complex suffering of terminally ill patients may lead to compassion fatigue, sadness, anger, and helplessness [38]. This emotional burden may affect professional performance, weaken nurses’ capacity to provide empathic care, and contribute to emotional distancing.

*N1: “When you are emotionally exhausted, it is difficult to be as empathetic as you want to be. You may find yourself becoming somewhat indifferent… I think reasonable scheduling is important for ensuring high-quality care.”*

Emotional support from colleagues, friends, and family was an important resource for coping with these challenges.

*N4: “Talking with colleagues who understand what you have experienced is healing…”*

*N5: “The companionship and encouragement of family and friends helped me get through that difficult time…”*

Peer support networks may therefore serve as an important protective factor against emotional exhaustion. Education also played a key role in maintaining nurses’ professional competence. Participants commonly emphasized the importance of continuous learning.

*N1: “We never stop learning. Keeping up to date and improving our skills are essential for providing the best care…”*

*N2: “This field is constantly developing, and we must keep pace with it. This is how we ensure the best care for patients…”*

These findings reflect nurses’ commitment to continuing professional development and support the value of systematic death education in improving death-coping competence [26,39,40].

The interviews also showed that nurses developed culturally embedded coping strategies in clinical practice. Some nurses used symbolic rituals to establish boundaries between work and daily life, such as changing uniforms or avoiding discussion of work after leaving the hospital.

*N3: “Some colleagues take a shower and change clothes immediately after providing end-of-life care, as a way to draw a boundary…”*

*N6: “Changing clothes means a change in identity. After work, I can stop reviewing the negative aspects of the workday…”*

Some nurses also incorporated culturally meaningful symbols into daily practice to reduce psychological stress associated with death.

*N7: “Some colleagues put amulets in their uniform pockets to bring good luck and ward off misfortune…”*

*N8: “We place apples at the nurses’ station, symbolizing a safe night shift without death-related events.”*

## Discussion

This study systematically assessed death-coping competence among geriatric nurses and further explored their experiences, attitudes, and care practices related to death and end-of-life care through semi-structured interviews. In the context of a cultural environment in which death remains a sensitive and often avoided topic, this study provides evidence for developing culturally sensitive death education strategies and targeted training programs. These findings may offer practical implications for improving the quality of geriatric end-of-life care. However, given that this study employed a single-center convenience sampling method, the generalizability of the findings may be limited.

### Death-coping competence at a moderate level

The total death-coping competence score among geriatric nurses in this study was 110.12 ± 24.35, with a mean item score of 3.93 ± 0.87, indicating a moderate level of death-coping competence. This finding is consistent with those reported in previous studies of Chinese geriatric nurses [3,41 7]. However, the score was lower than that reported in a Spanish study, in which 61.2% of 534 nurses reported a strong ability to cope with death [41]. This difference may be partly explained by cultural variations in attitudes toward death and end-of-life care. In many Western cultural contexts, death-related communication is often addressed more openly, and end-of-life care frequently emphasizes holistic care, quality of life, and quality of dying. In contrast, within traditional Chinese culture, death is often associated with misfortune, sorrow, and fear. As a result, death may be regarded not only as a taboo topic but also as a culturally sensitive issue, leading many people to avoid death-related discussions [36,42]. In addition, palliative care education and training in China remain relatively underdeveloped, and geriatric nurses may have limited knowledge and preparation for death-related care [43]. These factors may contribute to their relatively lower death-coping competence.

### Factors associated with death-coping competence among geriatric nurses

Multiple linear regression analysis showed that age, professional title, years of geriatric nursing experience, educational level, department, and previous participation in death education or related training were independently associated with death-coping competence among geriatric nurses (*p* < 0.05). These findings are generally consistent with previous research [44].

#### Age.

Age was a significant positive predictor of death-coping competence (*β* = 0.131, *p* = 0.023). Older nurses may have richer life experiences and a deeper understanding of life and death, which may enable them to view death more rationally and experience lower levels of death-related fear and avoidance. With increasing age and accumulated exposure to end-of-life care, nurses may gradually develop stronger self-efficacy in coping with patient death [39,45].

#### Professional title and nursing seniority.

Professional title was positively associated with death-coping competence (*β* = 0.241, *p* < 0.001). Nurses with higher professional titles are often more experienced in caring for terminally ill patients and communicating with their families. Their repeated exposure to grief, anxiety, and other emotionally challenging situations may help them develop more effective coping strategies [46]. In addition, nurses with higher professional titles may have more opportunities for continuing education, professional training, and exposure to advances in hospice and palliative care, which may further enhance their understanding of death and improve their coping competence. For nurses with lower professional titles or less clinical experience, systematic training and structured experience-sharing activities may help them develop a more positive and scientifically informed view of death.

#### Years of geriatric nursing experience.

Years of geriatric nursing experience were negatively associated with death-coping competence (*β* = −0.143, *p* = 0.033). This finding suggests that longer work experience does not necessarily lead to stronger death-coping competence. Nurses with 1–5 years of experience may still be adapting to clinical practice and may lack sufficient experience in end-of-life care, making it difficult for them to apply existing knowledge flexibly when facing patient death. Meanwhile, nurses with 11–15 years of experience may have been repeatedly exposed to the grief and emotional distress of terminally ill patients and their families. Without timely psychological support, this prolonged emotional burden may contribute to cumulative emotional exhaustion and reduced death-coping competence. This result indicates that the accumulation of work experience alone is insufficient. Psychological support, continuing education, and burnout prevention should be integrated into professional development programs for geriatric nurses.

#### Educational level.

Educational level was positively associated with death-coping competence (*β* = 0.140, *p* = 0.043). Nurses with higher educational attainment may have received more systematic training in death education, life ethics, and clinical decision-making. They may also have broader knowledge structures and stronger critical thinking skills, enabling them to analyze death-related events more rationally [46,47]. Therefore, encouraging nurses to pursue higher education and incorporating death education into continuing professional education may be important strategies for improving death-coping competence.

#### Department.

Department was independently associated with death-coping competence (*β* = 0.178, *p* = 0.007). ICU nurses showed higher death-coping competence than nurses working in medical and surgical wards. This may be related to the ICU work environment, where nurses are more frequently exposed to critical illness, emergencies, and patient death. Such repeated exposure may promote the development of adaptive coping mechanisms. In addition, the team-based care model in ICUs may provide stronger professional and emotional support. This finding is consistent with previous studies [37,48]. Training models and support systems used in ICUs may therefore provide useful references for improving death-coping competence among nurses in general wards.

#### Previous death education or related training.

Nurses who had received death education or related training had significantly higher death-coping competence than those without such training (*β* = 0.136, *p* = 0.032). Death education may not only address knowledge gaps but also promote changes in nurses’ attitudes toward death, helping them move from avoidance toward acceptance. Through approaches such as simulation training, case-based discussion, and reflective learning, nurses can acquire practical communication skills and emotion-regulation strategies, thereby improving their ability to cope with death-related care situations [49]. Hospitals should therefore provide continuous and systematic death education, especially in areas where nurses report limited knowledge, such as symptom management, psychological support, spiritual care, and communication with patients and families [46,50].

#### Other factors.

In this study, religious belief was not independently associated with death-coping competence (*p* > 0.05), which may be related to the high proportion of participants without religious affiliation (93.60%). Univariate analysis showed that previous involvement in end-of-life care for relatives or friends was marginally associated with death-coping competence (*t* = 1.872, *p* = 0.051). However, af*t*er this variable was included in the multiple linear regression model, its independent effect was not statistically significant (*p* = 0.169). This finding suggests that personal experiences with the death of relatives or friends may influence professional death-coping competence through interactions with other factors, such as age, death education, occupational experience, or social support, rather than acting as a direct independent predictor. This is partly consistent with previous research [33], indicating that the effect of personal death-related experiences on professional coping competence may be moderated by professional training and support resources. Future studies should further examine the interaction between personal end-of-life experiences and death education.

### Explorations on the implementation of death education strategies

Based on the quantitative findings and semi-structured interviews, this section further explores the challenges faced by geriatric nurses in the context of cultural taboos surrounding death. The following death education strategies, which incorporate characteristics of traditional Chinese culture, are proposed:

(1) **Strengthening systematic death education**

Given nurses’ knowledge gaps, limited training opportunities, and negative attitudes toward death care, departments should provide a supportive work environment and sufficient emotional support. Educational activities such as lectures, themed films, experience-sharing sessions, and literature discussions may help nurses deepen their understanding of death and the meaning of life and develop a more positive view of death.

(2) **Transforming traditional cultural resources into supportive coping resources**

Traditional cultural resources should be positively transformed and incorporated into death education. Culturally appropriate practices may help nurses constructively reinterpret death care. For example, symbolic objects or rituals may serve as psychological coping resources when used appropriately and voluntarily. More importantly, education should help nurses reframe end-of-life care not as a source of misfortune, but as a meaningful professional responsibility that preserves dignity, relieves suffering, and brings comfort to patients and families.

(3) **Integrating professional values with social values**

Caring for older adults and accompanying patients at the end of life can be framed as both a professional responsibility and a social virtue. Through education and training, nurses may strengthen their professional identity, reduce avoidance of death-related topics, and develop greater confidence in discussing and managing patient death. Providing dignified care before and after death should be understood as an important expression of nursing value.

(4) **Establishing ritualized practices and psychological boundaries**

Appropriate rituals before and after work may help nurses separate professional responsibilities from personal life and maintain psychological well-being. For example, changing out of the nursing uniform can serve as a symbolic transition from the professional role to personal life. Such practices may help nurses maintain work–life balance and reduce emotional spillover.

(5) **Optimizing nursing schedules and staffing arrangements**

Because death-coping competence was associated with age, years of experience, and death education, staffing arrangements should consider nurses’ age structure, clinical experience, exposure to patient death, and cultural beliefs. In departments with higher mortality, nursing managers should allocate staff according to professional title, qualifications, expertise, and experience. When patient admissions and deaths increase, timely technical and psychological support should be provided.

(6) **Strengthening team support and communication training**

Departments should foster supportive team relationships and use group discussions, interactive exercises, role-playing, and case-based learning to improve nurses’ communication skills and attitudes toward death. Strengthened team support may also help reduce emotional exhaustion and improve nurses’ confidence in end-of-life communication.

(7) **Establishing a continuous death education and support platform**

Hospitals should provide professional, emotional, and social support in the workplace and establish ongoing training systems for death education. Death-related theoretical models may be used to guide clinical practice, and psychological counseling or emotional support services should be made available to promote nurses’ mental health.

Overall, death education should shift from knowledge transmission alone to culturally embedded training. Through systematic education, positive transformation of cultural resources, boundary-setting rituals, team support, optimized staffing, and continuous professional development, nurses may better overcome death-related fear, strengthen death-coping competence, and provide high-quality, dignified end-of-life care for terminally ill patients and their families.

### Limitations

This study has several limitations. First, convenience sampling was used, and participants were recruited from geriatric departments in a single tertiary Grade A hospital in Kunming, China, which may limit the generalizability of the findings. In addition, nurses with strong fear or avoidance of death may have declined participation, potentially introducing selection bias. Second, the culturally responsive death education strategies proposed in this study have not yet been empirically tested. Future studies should evaluate their long-term effectiveness through randomized controlled trials or longitudinal intervention studies. Finally, although multiple potential influencing factors were included, unmeasured confounders, such as personality traits, family support, and organizational support, may still exist. Self-reported data may also be subject to social desirability bias. Future research should include larger and more diverse samples, adopt multicenter and longitudinal designs, and further examine the effectiveness and generalizability of the proposed educational strategies.

## Conclusion

This study systematically assessed death-coping competence and its associated factors among 203 geriatric nurses from a tertiary Grade A hospital in Kunming, China. The findings showed that nurses’ death-coping competence was at a moderate level, with a score rate of 54.19%. Age, educational level, department, professional title, years of geriatric nursing experience, and previous death education were identified as independent factors associated with death-coping competence. Based on the quantitative findings and semi-structured interviews, this study proposed culturally responsive death education strategies in the context of death-related taboos in traditional Chinese culture. Future training programs should consider nurses’ attitudes toward death, years of clinical experience, end-of-life care experience, cultural background, and religious beliefs. Implementing these strategies may help reduce negative emotional distress and burnout risk among geriatric nurses and improve the quality of end-of-life care. However, their long-term effectiveness should be further evaluated through multicenter, large-sample longitudinal studies and randomized controlled trials.

## Supporting information

S1 File Death-coping self-efficacy scale-English translation. (PDF)
