# Supplementary material for: Factors associated with death coping ability among geriatric nurses and development of culturally sensitive death education interventions
Source: PLoS One. 2026 Aug 25;21(8):e0356462. doi: 10.1371/journal.pone.0356462 (PMC13505890; doi:10.1371/journal.pone.0356462)
Supplement: Supplementary file 1 [file pone.0356462.s001.pdf]

Questionnaire Number:______________

Questionnaire on Attitudes towards Death and Coping Abilities of Geriatric Nurses

Dear teacher, hello!

First and foremost, we would like to express our gratitude for your participation in this survey. The information obtained from this survey is for personal use only. When filling out the questionnaire, please do not include your name. Your answers to the questions are not graded in any way. Please fill in according to your actual situation. We promise that except for use in our own research, we will not disclose any information in the questionnaire. Thank you very much for your participation and support in this survey!

Part One: General Information Survey

1. Your age: Years.
2. Gender:□Male □Female.
3. Your ethnicity: □Han ethnicity □Minority ethnic group.
4. Your religious belief is:

□None □Buddhism □Taoism and folk beliefs □Islam □Catholicism or Christianity

5. Your marital status:□Unmarried □Married □Divorced □Widowed
6. How many children do you have?

□0 children □1 child □2 children □3 or more children

7. Your highest educational attainment is:

□Associate degree □Undergraduate □Graduate student

8. Your working years: Years.
9. Your work department:□Internal medicine □Surgery □ICU
10. Methods of employment:□System of contract □Formal establishment
11. Your professional title:

□Nurse □Nurse practitioner □Nurse in charge □Associate chief nurse or above

12. Have you received death education or training: □Yes □No
13. Have you ever been involved in the end-of-life treatment of patients:□Yes □No
14. Have you been involved in the end-of-life management of a relative or friend:□Yes □No

Part Two: Death Attitude Scale

The following questions are about your attitude towards death. There are 5 answers on the right of each question, and there is no difference in the answer between right and wrong. Please quickly tick "√" according to your degree of agreement or disagreement with each statement. Thanks for your cooperation!

**Order number**	**The project**	**Agree completely**	**Agree**	**No opinion**	**Disagree**	**Strongly disagree**
1	Death is undoubtedly a terrifying experience.					
2	When I think about my own death, I become anxious and uneasy.					
3	I try my best not to think about death.					

4	I believe that after my death, I will go to heaven.					
5	Death will put an end to all my troubles.					
6	Death is natural, undeniable and inevitable.					
7	The certainty that everyone will eventually die troubles me.					
8	Death is the gateway to the ultimate paradise.					
9	Death allows me to escape from this terrifying world.					
10	Whenever the thought of death comes into my mind, I try to push it away.					
11	Death is the liberation from sorrow and suffering.					
12	I always try not to think about death.					
13	I believe that heaven is a better place than the present world.					
14	Death is a natural part of the process of life.					
15	Death is the union with God (deity, Buddha...) and eternal bliss.					
16	Death will undoubtedly bring about a brand new and glorious life.					
17	I neither fear nor welcome death.					
18	I have a strong fear of death.					
19	I completely avoid thinking about death.					
20	The question of whether there is life after death					

	has greatly troubled me.					
21	Death means the end of everything. This fact terrifies me.					
22	I look forward to being reunited with the ones I love after death.					
23	I regard death as the liberation from the suffering of this world.					
24	Death is merely a part of the life process.					
25	I regard death as a passage leading to eternal happiness and bliss.					
26	I try my best to avoid anything related to death.					
27	Death offers the soul a wonderful release.					
28	When facing death, what made me feel relieved was that I believed there would still be life after death.					
29	I regard death as the liberation from the burden of this life.					
30	Death is neither a good thing nor a bad thing.					
31	I have expectations for the life after death.					
32	The uncertainty of what might happen after death makes me worried.					

Part Three:Death Coping Ability Scale

Instructions: Please rate your agreement or disagreement with each item on a scale of 1 to 7. 1 indicates "completely disagree", 4 indicates "neutral", and 7 indicates "completely agree".

1 2 3 4 5 6 7

Completely disagree Neutral Completely agree

Serial Number	Item	1-7
1	I have a positive view of death and the process of dying.	
2	Death can be dealt with safely.	
3	I am aware of the range of services offered by the funeral home company.	
4	I'm aware of many options for handling the body.	
5	I am aware of all the manifestations that express human emotions of sadness.	
6	Knowing that I will eventually die has no effect on my life or my actions.	
7	I was prepared for death.	
8	I was prepared for the end-of-life process.	
9	I know where my fear of death lies.	
10	I am familiar with the preparatory matters for the funeral ceremony.	
11	Recently, I've come to realize that thinking about death is not at all (scary).	
12	Recently, my attitude towards life has changed.	
13	I can express my fear of death.	
14	I can clearly sense the approach of death and the end of life.	
15	I am making the most of my present life.	
16	The quality of my life is more important than its duration.	
17	I can talk about my death with my family and friends.	
18	I know whom to contact when death occurs.	

19	I will be able to cope with future losses (such as people, things, etc.)	
20	I feel capable of coping with the death of someone close to me.	
21	I know how to listen to others, including listening to terminally ill patients.	
22	I know how to talk to children about death.	
23	If necessary, I will take the time to be with terminally ill patients.	
24	I can assist people in their thoughts and emotions regarding death and the end of life.	
25	I can talk to my friends or family about his/her death.	
26	I was able to ease the anxiety of those around me when they talked about death or dying	
27	I was able to communicate with the dying	
28	Before I or someone else dies, I can tell them how much I love them.	
